# The preceding hyponatremia is a useful hallmark for the diagnosis of HHV-6 encephalitis after allogeneic hematopoietic stem cell transplantation

**DOI:** 10.1038/s41409-022-01843-y

**Published:** 2022-10-15

**Authors:** Shota Yoshida, Takahide Ara, Kohei Okada, Yuto Mori, Shihori Tsukamoto, Naoki Miyashita, Kohei Kasahara, Ko Ebata, Junko Iwasaki, Shojiro Takahashi, Akio Shigematsu, Koichiro Minauchi, Naoki Kobayashi, Masahiro Ogasawara, Masahiro Imamura, Takanori Teshima, Shuichi Ota

**Affiliations:** 1 Department of Hematology, Sapporo Hokuyu Hospital, Sapporo, Japan; 2 Department of Hematology, Hokkaido University Hospital, Sapporo, Japan; 3 Department of Hematology, Kushiro Rosai Hospital, Kushiro, Japan

**Keywords:** Infectious diseases, Risk factors

## To the Editor:

Human herpes virus-6 (HHV-6) encephalitis is one of the life-threatening complications after allogeneic hematopoietic stem cell transplantation (allo-HSCT). Early diagnosis and intervention are important for the prevention of poor prognosis and sequelae [1–3]. However, there are no specific neurological symptoms for HHV-6 encephalitis and polymerase chain reaction (PCR) testing of cerebrospinal fluid (CSF) for detection of HHV-6 may be hesitated at early after allo-HSCT due to cytopenia or hemorrhagic tendency [4]. Therefore, the diagnosis of HHV-6 encephalitis remains problematic.

A recent systematic review suggested the association of HHV-6 encephalitis and hyponatremia [5]. However, since most case reports did not specify the timing of onset of hyponatremia and HHV-6 encephalitis, it is unclear whether the preceding hyponatremia is a useful hallmark for the diagnosis of HHV-6 encephalitis. In order to address this issue, we retrospectively reviewed the dynamics of serum sodium levels and the incidence of HHV-6 encephalitis in 134 consecutive adult patients aged 18 or older who underwent allo-HSCT between April 2018 and December 2020 at Sapporo Hokuyu Hospital in Japan. This study was approved by the institutional review board of Sapporo Hokuyu Hospital.

HHV-6 encephalitis was diagnosed if the patient satisfied all of following criteria: (1) presence of central nerve system (CNS) dysfunction, which was defined as the presence of disorientation as to time and place, loss of consciousness, memory loss or convulsions, (2) a positive PCR result for HHV-6 DNA in CSF, and (3) absence of other identified causes of CNS dysfunction [2, 6]. The serum sodium levels were checked at least three times a week. Cerebrospinal PCR test for HHV-6 DNA were performed as soon as possible when CNS dysfunction appeared in allo-HSCT recipients. The diagnosis date of HHV-6 encephalitis was defined as the testing day on which PCR result for HHV-6 DNA became positive firstly. Based on the previously reported serum sodium levels at the onset of HHV-6 encephalitis, hyponatremia was defined as 130 mEq/L or less [5, 7]. In patient and transplant characteristics analysis, Fisher’s exact test was used to compare categorical values and Mann–Whitney U test was used to compare continuous values. The cumulative incidence of HHV-6 encephalitis and hyponatremia were estimated by Gray’s method while treating death as a competing risk. In the risk factor analysis, the Fine-Gray proportional hazard regression analysis was used, treating hyponatremia as a time-dependent covariate, in addition to the previously reported risk factors [4, 6]. Factors associated with at least borderline significance (*P* < 0.10) in the univariate analysis were subjected to a multivariate analysis. To assess the diagnostic values of each finding, we calculated sensitivity, specificity, positive predictive value (PPV), negative predictive value (NPV), likelihood ratio of a positive test result (LR+), likelihood ratio of a negative test result (LR−), and diagnostic odds ratio (DOR) [8]. Statistical significance was defined as a two-tailed *P* value < 0.05. All statistical analyses were performed with EZR ver. 1.52 (Jichi Medical University Saitama Medical Center), which is a graphical user interface for R (The R Foundation for Statistical Computing, Vienna, Austria) [9].

One hundred thirty-four patients with a median age 53 years (range, 19–72) included 59 acute myeloid leukemia, 31 acute lymphoblastic leukemia, 22 myelodysplastic syndrome/myeloproliferative neoplasm, 14 malignant lymphoma, 3 adult T-cell leukemia/lymphoma, and 5 other hematological diseases (Supplemental Table 1). Of these patients, 29, 45, and 60 patients underwent bone marrow transplantation, peripheral blood stem cell transplantation (PBSCT), and cord blood transplantation (CBT), respectively. Most patients (80.6%) received myeloablative conditioning regimens. The classification of graft-versus-host disease (GVHD) prophylaxis regimens and donor sources were summarized in Supplemental Table 2. In summary, tacrolimus (TAC) alone and TAC with mycophenolate mofetil (MMF) were mainly performed in CBT and post-transplantation cyclophosphamide with TAC and MMF were performed only in human leukocyte antigen-haploidentical PBSCT.

**Fig. 1:**
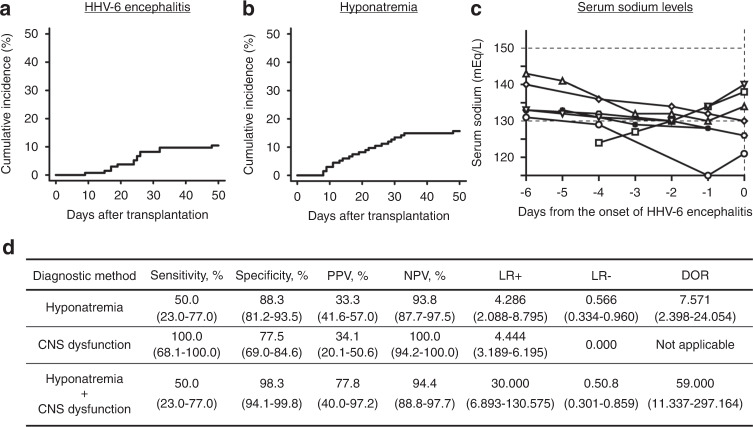
The preceding hyponatremia is a useful hallmark for the diagnosis of HHV-6 encephalitis after allogeneic hematopoietic stem cell transplantation. **a** Cumulative incidence of HHV-6 encephalitis until 50 days after allo-HSCT. **b** Cumulative incidence of hyponatremia until 50 days after allo-HSCT. **c** The serial changes of serum sodium levels a week before the onset of HHV-6 in each patient with hyponatremia. **d** Diagnostic accuracy index of hyponatremia, CNS dysfunction, and the coexistence of hyponatremia and CNS dysfunction for HHV-6 encephalitis. 95% confidence intervals is shown in parentheses.

A total of 14 (10.4%) patients developed HHV-6 encephalitis at a median of 24 days (range, 9–48 days) after allo-HSCT (Fig. 1a). All HHV-6 encephalitis developed within 50 days after allo-HSCT and 21 (15.7%) patients developed hyponatremia during this period with a median of 19 days (range, 8–48 days) (Fig. 1b). The syndrome of inappropriate secretion of antidiuretic hormone (SIADH) is the most common definitely documented pathogenesis of hyponatremia after allo-HSCT [10]. Although not all cases had been tested to formally diagnose SIADH, 2 of 14 patients with HHV-6 encephalitis and 1 out of 120 patients without HHV-6 encephalitis were diagnosed with SIADH. In univariate analysis, CBT (HR 4.748, 95% CI: 1.087–10.490, *P* = 0.029), GVHD prophylaxis with TAC alone (HR 8.064, 95% CI: 1.537–13.830, *P* = 0.005), and hyponatremia (HR 9.615, 95% CI 2.946–31.380, *P* = 0.0001766), treating as a time-dependent covariate, were significantly correlated with the development of HHV-6 encephalitis. In multivariate analysis, hyponatremia (HR 9.501, 95% CI 2.534–35.620, *P* = 0.0008399) was identified as a significant risk factor for the development of HHV-6 encephalitis (Supplemental Table 3).

Furthermore, we investigated whether hyponatremia could precede the onset of HHV-6 encephalitis. In the 14 patients who developed HHV-6 encephalitis, seven (50.0%) patients had preceded hyponatremia within a week before the diagnosis of HHV-6 encephalitis (Fig. 1c). On the other hand, 14 (66.7%) of 21 patients who had hyponatremia did not develop HHV-6 encephalitis. Thus, the sensitivity, specificity, PPV, NPV, LR+, and LR− of hyponatremia for the diagnosis of HHV-6 encephalitis were 50%, 88.3%, 33.3%, 93.8%, 4.286, and 0.566, respectively (Fig. 1d). Allo-HSCT recipients may develop CNS dysfunction due to many other factors than HHV-6 encephalitis [11]. In fact, 27 (65.9%) of 41 patients with CNS dysfunction had other causes than HHV-6 encephalitis in this study. The sensitivity, specificity, PPV, NPV, LR+, and LR− of CNS dysfunction for the diagnosis of HHV-6 encephalitis were 100%, 77.5%, 34.1%, 100.0%, 4.444, and 0.000, respectively (Fig. 1d). Therefore, CNS dysfunction alone was insufficient for the diagnosis in terms of the specificity. Interestingly, the sensitivity, specificity, PPV, NPV, LR+, and LR− of the coexistence of CNS dysfunction and hyponatremia for the diagnosis of HHV-6 encephalitis were 50%, 98.3%, 77.8%, 94.4%, 30.000, and 0.508, respectively (Fig. 1d). Moreover, this combination remarkably increased DOR from 7.571 to 59.000 compared to the hyponatremia alone, indicating that the coexistence of CNS dysfunction and hyponatremia could improve the diagnostic accuracy of HHV-6 encephalitis (Fig. 1d).

Our study indicated that the preceding hyponatremia specifically observed within a week before the onset of HHV-6 encephalitis, consistent with the previous report [7]. In addition, the coexistence of hyponatremia and CNS dysfunction after allo-HSCT strongly suggests HHV-6 encephalitis and this combination may prompt to assess the CNS disease by lumbar puncture and lead to the early initiation of anti-HHV-6 therapies with more efficiently.

The current study had several limitations. First, it is still unclear whether the cutoff value of 130 mEq/L or less is appropriate for the differential diagnosis of HHV-6 encephalitis due to the retrospective and small number study. Second, we did not evaluate other factors that may affect serum sodium level such as fluid replacement, diuretic drugs, vomiting, and diarrhea [12].

In conclusion, our study suggests the possibility of HHV-6 encephalitis significantly increases when appearing CNS dysfunction following hyponatremia after allo-HSCT. When hyponatremia emerge after allo-HSCT, we should monitor carefully about the symptoms of HHV-6 encephalitis. In future, a large-scale prospective studies are warranted to confirm our findings.

## Supplementary information

Supplemental Tables

## Data Availability

The datasets generated during and/or analyzed during the current study are available from the corresponding author on reasonable request.
