# Supplementary material for: The preceding hyponatremia is a useful hallmark for the diagnosis of HHV-6 encephalitis after allogeneic hematopoietic stem cell transplantation
Source: Bone Marrow Transplant. 2022 Oct 15;58(1):97–9. doi: 10.1038/s41409-022-01843-y (PMC9812762; doi:10.1038/s41409-022-01843-y)
Supplement: Supplementary file 1 — Supplemental Tables [file 41409_2022_1843_MOESM1_ESM.pdf]

Supplemental Table 1. Patient and transplant characteristics

	All patients (N=134)	HHV-6 encephalitis		*P* Value
		Positive (N=14)	Negative (N=120)	
Median age (years) [range]	53 [19-72]	46 [28-68]	52 [20-70]	0.951
Patient Age				
<50 years	57	8	49	0.266
≧50 years	77	6	71	
Gender				
Male	88	10	78	0.771
Female	46	4	42	
Disease				
AML	59	9	50	0.167
ALL	31	0	31	
MDS/MPN	22	3	19	
Malignant Lymphoma	14	1	13	
Adult T-cell leukemia/lymphoma	3	0	3	
Others	5	1	4	
Refined-DRI				
Very High/High	76	11	65	0.095
Intermediate	58	3	55	
Donor source				
Related BM or PB, HLA match	16	0	16	0.145
Related BM or PB, HLA mismatch	32	1	31	
Unrelated BM or PB, HLA match	13	2	11	
Unrelated BM or PB, HLA mismatch	13	1	12	
CB	60	10	50	
Conditioning regimen				
MAC	108	11	97	0.735
RIC	26	3	23	
GVHD prophylaxis				
TAC alone	17	5	12	**0.018**
Multiple drugs combination	117	9	108	

AML acute myeloid leukemia, *ALL* acute lymphoblastic leukemia, *BM* bone marrow, *CB* cord blood, *DRI* disease risk index, *GVHD* graft-versus-host disease, *HLA* human leukocyte antigen, *MAC* myeloablative conditioning, *MDS* myelodysplastic syndrome, *MPN* myeloproliferative neoplasm, *PB* peripheral blood stem cell, *RIC* reduced-intensity conditioning, *TAC* tacrolimus.

Supplemental Table 2. Classification of GVHD prophylaxis regimens

	Total allo-HSCT (N=134)		
	BMT (N=29)	PBSCT (N=45)	CBT (N=60)
TAC+sMTX (N=44)	25	6	13
TAC+sMTX+ATG (N=10)	4	6	0
TAC+MMF (N=33)	0	1	32
TAC alone (N=17)	0	2	15
PTCY+TAC+MMF (N=30)	0	30	0

Allo-HSCT allogeneic hematopoietic stem cell transplantation, *ATG* anti-thymocyte globulin, *BMT* bone marrow transplantation, *CBT* cord blood transplantation, *MMF* mycophenolate mofetil, *PBSCT* peripheral blood stem cell transplantation, *PTCY* post-transplantation cyclophosphamide, *sMTX* short-term methotrexate, *TAC* tacrolimus.

Supplemental Table 3. Univariate and multivariate analysis of risk factors for the cumulative incidence of HHV-6 encephalitis

	Univariate analysis		Multivariate analysis	
	Hazard ratio (95%CI)	*P* Value	Hazard ratio (95%CI)	*P* Value
Male Recipient	0.207 (0.412-4.149)	0.649	-	-
Refined-DRI：<br>Very High / High	3.157 (0.854-10.470)	0.076	2.959 (0.811-10.800)	0.1005
HLA-Mismatched Donor	0.548 (0.403-7.471)	0.459	-	-
CBT	4.748 (1.087-10.490)	**0.029**	2.457 (0.658-9.179)	0.1811
MAC	0.033 (0.254-3.113)	0.856	-	-
TAC alone	8.064 (1.537-13.830)	**0.005**	1.981 (0.485-8.090)	0.341
ES / PIR	3.277 (0.909-7.210)	0.07	1.130 (0.307-4.165)	0.8539
Hyponatremia	9.615 (2.946-31.380)	**0.0001766**	9.501 (2.534-35.620)	**0.0008399**

CBT cord blood transplantation, *DRI* disease risk index, *ES* engraftment syndrome, *HLA* human leukocyte antigen, *MAC* myeloablative conditioning, *PIR* pre-engraftment immune reaction, *TAC* tacrolimus.
